# Primary care pediatricians’ attitudes and practice towards HPV vaccination: A nationwide survey in Italy

**DOI:** 10.1371/journal.pone.0194920

**Published:** 2018-03-29

**Authors:** Francesco Napolitano, Monica Navaro, Luigi Vezzosi, Gabriella Santagati, Italo Francesco Angelillo

**Affiliations:** Department of Experimental Medicine, University of Campania “Luigi Vanvitelli”, Naples, Italy; Cincinnati Children’s Hospital Medical Center, UNITED STATES

## Abstract

This national online cross-sectional survey in Italy assessed primary care pediatricians’ (PCPs) attitudes and practices regarding Human papillomavirus (HPV) vaccination and the contribution of several characteristics. The questionnaire was distributed from September 2016 to June 2017 to a random sample of 640 PCPs by email via an internet-link leading to a web-based survey platform (Lime Survey). Only 18.4% of PCPs always recommend the HPV vaccine to 11–12 year old boys. PCPs with longer practice activity, working in solo practice, always recommended the HPV vaccine to 11–12 year old girls, and believed that the vaccine was effective for boys were more likely to always recommend the HPV vaccine. PCPs working in a Region where the vaccination was actively recommended and provided free of charge to 11–12 year old boys had higher odds of recommending vaccination. More than two thirds of PCPs (77.4%) always recommend the HPV vaccine to 11–12 year old girls. PCPs who believed that the vaccine was effective for girls and safe in both boys and girls, who always talk with patients of 11–18 years or their parents about HPV infection and vaccination, and who obtain vaccine information from scientific journals were more likely to always recommend the vaccine. PCPs should employ evidence-based educational strategies in order to achieve a better coverage and to reduce the morbidities and mortality of diseases associated with HPV.

## Introduction

Human papillomavirus (HPV) infection is still a growing public health threat worldwide due to the morbidity, mortality, and costs related to cervical, vulvar, anal, penile, head, and neck cancers [1,2]. It is well known that the principal risk factors are related to the sexual behavioral such as early age of sexual debut, numbers of lifetime sexual partners, and inconsistent condom use [3–6]. Clinical trials have demonstrated the safety, efficacy, and immunogenicity of the HPV vaccine [7,8] and, therefore, the implementation of high-quality prophylactic programs is a priority because it has the potential to reduce the rates of HPV infection and associated diseases. Despite the HPV vaccination in Italy at the time of this study was available within the public health service at no cost for girls and boys aged 11 or 12 years and for girls through age 26 years and boys through age 21 years if not previously vaccinated, rates of vaccination uptake still remain well below national public health goals [9]. Indeed, the vaccination uptake in 11-year old girls is 62.1% for the 2003 birth cohort, whereas no data for boys is available [10]. In Italy, parents represent the legal decision-makers of their children <18 years old and, therefore, their knowledge and beliefs have an impact on the vaccination coverage rate [11–13]. Moreover, reasons to HPV vaccination rate incompliance includes attitudes and recommendations of health-care providers, limited access to health-care services, poor knowledge and awareness about HPV and cervical cancer risk, and concerns about the safety and efficacy of the vaccine. Therefore, for the successful implementation of the HPV vaccination, health care providers, particularly in primary care, are a key component in promoting the vaccination and its acceptability, since they have general influence over the health behavior of their patients. While there has been published research on the levels of knowledge and awareness about HPV in different groups [14–18] also in this geographic area [19–21], relatively little public health investigation has focused on primary care pediatricians (PCPs) [22–25]. Understanding their perception and practice in relation to HPV vaccination is crucial because PCPs could be a key element in the promotion of HPV prevention and the design of effective educational efforts. In order to address this notable gap in the literature, the aims of the present national cross-sectional survey in Italy were to assess the PCPs’ attitudes and practices regarding HPV vaccination and the relative contribution of several characteristics.

## Materials and methods

### Participants

The online survey was conducted from September 2016 to June 2017 performing a multi-stage sampling. A random sample of Local Health Units (LHUs), which organize, plan, and provide health care services for the community closer to where people live, was selected across Italy. The Head of the selected LHUs received a letter explaining the purpose of the study, and after consent the research team received email-addresses and phone numbers of potential participants. Then, a random sample of 640 PCPs was recruited from the list provided by each selected LHU.

The sample size was calculated based on the estimation that 60% of the PCPs recommend HPV vaccination, a 95% confidence interval, and an error of 0.05. Therefore, the minimum number of pediatricians required was estimated at 360. In addition, accounting for a response rate of 55%, the minimum number of participants required was estimated as 640.

### Procedures

The study instrument was a questionnaire distributed to the sample by e-mail via an internet-link leading to a web-based survey platform (Lime Survey). The e-mail contained a cover letter with the lead investigator’s name and contact details, and explained, as well as on the web-based surveys platform, the background, the objectives, and the methodology of the study. The participants were invited to self-administer a confidential survey by clicking on a unique URL for each respondent, which remained active for completion until June 30, 2017. The study was absolutely voluntary and the cover letter specified that personal data were collected and analyzed anonymously, and each personal number of the URL link was stored on a secure server to know those who responded. Participants were informed before proceeding to the survey that sending back the anonymous questionnaire, they gave the informed consent to participate, and they were able to exit the survey at any stage. As the survey was completed anonymously, by clicking the “submit” button at the end of the survey, it was not possible to withdraw individual respondents’ data. In an effort to maximize the response rate, eight subsequent additional e-mail reminders at 4-week intervals were sent to the PCPs who had not already responded soliciting them to complete the questionnaire. Finally, non-responders were personally phone called by the research team soliciting their participation. There was no monetary compensation to participate in this study.

### Survey instrument

Prior to the start of the study, the questionnaire was pre-tested and piloted involving a sample of 25 physicians, not included in the final sample, to ensure clarity and ease of administration. The complete questionnaire in Italian and English are found in S1 and S2 Files. Based on respondents’ recommendations, some changes were incorporated to simplify and improve the final questionnaire. The completion time of the questionnaire was estimated to be approximately 10 minutes and it encompassed 46 items covering the following four main areas: 1) personal (age, gender, marital status), professional (year of graduation, specialty), and work practice characteristics (geographic area of activity, number of years in practice, practice size, number of patients, number of patients in the target age group of 11–12 years) of the respondents; 2) attitude toward the effectiveness and safety of the HPV vaccination; 3) behaviors regarding the collection of information on the sexual habits of patients in the target age group of 11–12 years; and 4) sources of information on HPV infection and vaccination. The survey included open-ended responses, categorical responses, such as yes/no/do not know, 10-points Likert scale with higher values corresponded to stronger attitude, 5-points Likert scale with response ranging from never to always, and selection of responses from a list either through drop-down menus that allowed single responses or check boxes that allowed multiple responses. To avoid the possibility of missing data, all survey questions were compulsory such that each item had to be answered before moving on the next item. At the conclusion of the survey, participants were given the opportunity to provide any free comment they had on the survey.

### Ethical statement

The Institutional Review Board of the Teaching Hospital of the University of Campania “Luigi Vanvitelli” approved all study procedures.

### Data analysis

Statistical analysis of the data (S3 Dataset) was undertaken using the Stata software version 10.1 [26]. First, descriptive statistics including frequency distribution, mean, standard deviation, and range were calculated to characterize respondents and their responses. Second, univariate analysis, independent *t* or chi-square tests, as appropriate, were carried out to evaluate predictors of the different outcomes of interest, after which the candidates for the multivariate regression analysis were selected. Only characteristics with *p*-values below or equal 0.25 in univariate analyses with the outcomes were included in the regression models. Third, multivariable logistic regression models for dichotomous outcomes were constructed to determine independent associations between different covariates and the following outcomes of interest: profile of PCPs who often/always collect information on the sexual habits of adolescents of 11–18 years (Model 1), profile of PCPs who always recommend the vaccine to 11–12 year old boys (Model 2), and profile of PCPs who always recommend the HPV vaccine to 11–12 year old girls (Model 3). PCP socio-demographic and professional independent variables included in all models were the following: age (continuous, in years), gender (male = 0; female = 1), number of years since degree (continuous), number of years in practice (continuous), number of hours worked per week (continuous), practice type (solo = 0; group = 1), always talk about HPV infection and its vaccination with patients of 11–18 years or their parents (no = 0; yes = 1), sources of information about the HPV vaccination (none = 0; scientific journals = 1; other = 2), and need to receive additional information about the HPV vaccination (no = 0; yes = 1). Moreover, the variable number of patients (continuous) was included in Model 1. The variable believe that the HPV vaccine was safe in both boys and girls (continuous) was included in Models 2 and 3. The following independent variables were also included in Model 2: number of 11–12 year old boys patients (continuous), working in a Region where the HPV vaccination was actively recommended and provided free of charge to 11–12 year old boys (no = 0; yes = 1), believe that the HPV vaccine was effective for boys (continuous), and having always recommended the HPV vaccine to 11–12 year old girls (no = 0; yes = 1). Finally, the variables number of 11–12 year old girls patients (continuous), and believe that the HPV vaccine was effective for girls (continuous) were included in Model 3. Stepwise selection was used to determine the final models and the significance level for variables entering in the models was set at 0.2 and for removing at 0.4. Multivariate logistic regression models produced adjusted odds ratios (ORs) and their corresponding 95% confidence intervals (CIs) as measures of association between predictors and outcomes of interest. All statistical tests were two-tailed, and *p*-value below or equal 0.05 was considered to indicate a statistically significant difference.

## Results

### Participant characteristics

Of the 640 PCPs selected, a total of 234 participants completed the online questionnaire for a response rate of 36.5%. Table 1 summarized the socio-demographic and professional characteristics of the survey participants. Two third of the sample were women (64.5%), the mean age was 55.7 years, mean length of practice activity was 21.2 years, nearly one-third practiced in a group setting (31.2%), and provided care to a mean of 904 patients. Regarding non-respondents PCPs, 67.3% were women, the average age was 57.8 years, the mean length since degree was 31.2 years, and no significant differences has been observed with the respondents.

**Table 1:** Main characteristics of the study population.

Characteristic	n	%
*Gender*		
Male	83	35.5
Female	151	64.5
*Age*, *years*	55.7±7.6(35–71)*	
*Number of years since degree*	29.5±7.6(9–46)*	
*Number of years in practice*	21.2±9.3(1–41)*	
*Practice type*		
Solo	161	68.8
Group	73	31.2
*Working in a Region where the HPV vaccination was actively recommended and provided free of charge to 11–12 year old boys*		
Yes	53	22.6
No	181	77.4
*Number of hours worked per week*	32±8.8(5–60)*	
*Number of patients*	904.3±233.6(130–1400)*	
*Number of 11–12 year old girls patients*	80.3±64.4(3–500)*	
*Number of 11–12 year old boys patients*	77.1±64.6(5–584)*	

*Mean±Standard deviation (range)

### Attitudes towards the HPV vaccination

Regarding the respondents’ attitude towards HPV vaccination, all the three statements received a high value. Indeed, almost all PCPs believed that the vaccine is effective in preventing HPV-related diseases in boys (92.3%) and in girls (97.9%), with an overall mean value of 8.7 out of a maximum score of 10. Moreover, 97.4% of PCPs felt safe the vaccine in both boys and girls, with an overall mean value of 8.8 out of a maximum score of 10.

### Behaviors towards the HPV vaccination

During health maintenance visits, only 10.3% of the PCPs indicated that they collect often/always information on the sexual habits of their patients and a quarter (25.6%) never did it. Only 39.7% and 29.1% talked respectively often and always about HPV infection and its vaccination with patients of 11–18 years or their parents. Findings from the multivariable logistic regression analyses with stepwise elimination procedure of factors associated with the different outcomes of interest are shown in Table 2. In Model 1, after adjustment for covariates, only one variable remains significantly associated with PCPs who often/always collect information on the sexual habits of adolescents of 11–18 years. PCPs who always talk about HPV infection and its vaccination with patients of 11–18 years or their parents were more likely to often/always collect this information (OR = 3.79; 95% CI = 1.58–9.09). Regarding the HPV vaccination practices, more than half (58.9%) of the sample indicated that they recommend the HPV vaccine with option ranging from rarely to always to 11–12 year old boys, and a total of 18.4% always recommend it. The final multivariable logistic regression model predicting binary vaccine recommendation outcome indicated that longer practice activity (OR = 1.12; 95% CI = 1.01–1.26), working in solo practice (OR = 0.29; 95% CI = 0.09–0.87), always recommendation of the HPV vaccine to 11–12 year old girls (OR = 12.38; 95% CI = 2.33–65.64), and believe that the vaccine was effective for boys (OR = 1.61; 95% CI = 1.15–2.26) were independently associated with this behavior. Additionally, PCPs working in a Region where the vaccination was actively recommended and provided free of charge to 11–12 year old boys had 18 times higher odds of recommending vaccination compared to those who practiced in a Region where it was not actively recommended (OR = 18.2; 95% CI = 6.36–51.96) (Model 2). Among PCPs who indicated that they recommend the vaccine to 11–12 year old boys, the major reasons were that the vaccine offered the opportunity of preventing HPV-related diseases (73%), that it is effective (51.4%), and it is safe (51.3%). On the other hand, PCPs who did not recommend often/always the vaccination justified their position by the fact that in their Region the vaccine for this group was not actively recommended (88.7%). The other main reasons were the lack of time to talk with their patients and parents about HPV infection and vaccination (10.6%) and the concerns about vaccine efficacy or side effects (5.6%). Among PCPs who recommend the HPV vaccine, 78% indicated that there are boys who refused to be vaccinated. More frequently reported reasons for this behavior were that they believed they were not at risk of getting HPV infection (35.9%), concerns regarding the side effects (30.7%), costs (26.5%), and insufficient knowledge about HPV infection (25.9%). Moreover, only 9.4% of PCPs always recommended the HPV vaccine to 13–18 year old boys not previously vaccinated.

**Table 2:** Multivariable logistic regression analysis of factors predicting the different outcomes of interest.

Variable	OR	SE	95% CI	*p* value
**Model 1.** PCPs who often/always collect information on the sexual habits of adolescents of 11–18 years				
Log likelihood = -71.53, χ2 = 11.69 (2 df), p = 0.002				
Always talking about HPV infection and its vaccination with patients of 11–18 years or their parents	3.79	1.69	1.58–9.09	0.003
Women	1.92	1.02	0.67–5.48	0.145
**Model 2.** PCPs who always recommend the vaccine to 11–12 year old boys				
Log likelihood = -73.39, χ2 = 76.47 (5 df), p<0.0001				
Working in a Region where the vaccination was actively recommended and provided free of charge to 11–12 year old boys	18.2	9.73	6.36–51.96	<0.001
Having always recommended the HPV vaccine to 11–12 year old girls	12.38	10.53	2.33–65.64	0.003
Believe that the HPV vaccine was effective for boys	1.61	0.28	1.15–2.26	0.005
Longer practice activity	1.12	0.06	1.01–1.26	0.028
Solo practice	0.29	0.16	0.09–0.87	0.028
Younger PCPs	0.93	0.06	0.82–1.07	0.318
Always talking about HPV infection and its vaccination with patients of 11–18 years or their parents	1.49	0.67	0.61–3.61	0.375
**Model 3.** PCPs who always recommend the vaccine to 11–12 year old girls				
Log likelihood = -78.29, χ2 = 93.8 (8 df), p<0.0001				
Always talking about HPV infection and its vaccination with patients of 11–18 years or their parents	21.06	18.81	3.65–121.27	0.001
Believe that the HPV vaccine was safe in both boys and girls	1.98	0.46	1.26–3.12	0.003
Sources of information				
Scientific journals	1*			
Other	0.28	0.14	0.11–0.73	0.009
Believe that the HPV vaccine was effective for girls	1.67	0.36	1.09–2.54	0.018
Group practice	2.52	1.21	0.98–6.46	0.054
Lower number of 11–12 years old girls patients	0.99	0.03	0.98–1.01	0.072
Women	2.07	0.87	0.91–4.72	0.084
Need to receive additional information about the HPV vaccination	1.54	0.64	0.67–3.49	0.304

* Reference category

Almost all the sample (98.7%) indicated that they recommend the HPV vaccine with option ranging from rarely to always to 11–12 year old girls, and a total of 77.3% always recommend it. On the basis of univariate findings, the multivariable logistic regression model for the recommendation of the vaccination to this group included eight variables. The final multivariable model predicting this outcome indicated that PCPs who believed that the vaccine was effective for girls (OR = 1.67; 95% CI = 1.09–2.54) and safe in both boys and girls (OR = 1.98; 95% CI = 1.26–3.12), those who always talk with patients of 11–18 years or their parents about HPV infection and vaccination (OR = 21.06; 95% CI = 3.65–121.27) were more likely to recommend the vaccine to 11–12 year old girls. Moreover, compared with PCPs who obtain vaccine information from scientific journals, those who used other sources were less likely (OR = 0.28; 95% CI = 0.11–0.73) to recommend the vaccine (Model 3). The reasons more frequently reported by PCPs who did not recommend often/always the vaccine for 11–12 year old girls were lack of time to talk about HPV infection and vaccination (60%), concern that vaccination could increase the high-risk sexual behaviors (25%), concern about the efficacy (22.7%) and safety (15%) of the vaccine. Instead, the reasons more frequently reported by respondents who often/always recommend the HPV vaccine were that in their Region it was actively recommended and provided free of charge to girls (75.7%) and that the vaccine can prevent the HPV-related cancers (69.2%).

Among survey respondents who recommended the HPV vaccine to girls aged 11–12 years, 71.4% reported that some of them refused to be vaccinated. The most frequent reasons cited by the respondents were concerns regarding the adverse side effects (46.7%), that patients did not believe in the usefulness of vaccination (40.3%), and felt they were not at risk for HPV infection (37%). Furthermore, 51.7% always recommended the vaccine to 13–18 year old girls not previously vaccinated.

Overall, 77.4% and 80.8% of PCPs reported having received information about HPV infection and the relative vaccination, respectively. Scientific journals and educational courses/meetings were the most trusted sources by the PCPs in order to obtain information (76.2% and 74.6% for the infection and 79.9% and 83.6% for the vaccination), followed by colleagues (13.2% for the infection and 9.4% for the vaccination), and pharmaceutical representatives (10.6% for the infection and 6.6% for the vaccination). About half (42.3%) of PCPs reported that they felt the need to receive additional information about the HPV infection and more than half (51.3%) about the vaccine.

## Discussion

This study characterized the attitudes and practices of a large sample of PCPs in Italy regarding HPV vaccination and determined which factors were associated and it has important implication for the program planning and implementation regarding the vaccination.

In this nationally representative sample of PCPs, respectively 77.3% and 18.4% reported that they always recommend the HPV vaccine to girls and boys aged 11–12. In the literature, previous results from the United States highlighted that 57% [27] and 60% [24] PCPs recommended HPV vaccines to girls at 11–12 year-old, 67% routinely [28] and 52% strongly [24] recommend at 11–12 year old boys, and 47.9% always to 11–12 year old [29]. A study in France on a sample of General Practitioners, since teenage girls no longer consult pediatricians, reported that 45.6% always recommend the HPV vaccine to girls aged 11–14 [30]. The routinely recommendation rates in the present study are very low and alarming, for boys, since HPV infection in sexually active homosexual and heterosexual men is problematic also because they can transmit HPV to their partners. Moreover, the fact that the proportion of those who accepted to receive the vaccine in the present study was below expectations, since 78% and 71.4% of 11–12 year old boys and girls did not accept to be vaccinated, is not encouraging from a public health point of view. Indeed, vaccination of boys is likely to provide benefit to girls through herd immunity and low recommendation and poor adherence puts adolescents at risk for HPV-related sequelae [31]. Therefore, public health efforts are strongly needed to implement strategies to encourage PCPs to recommend HPV vaccination, as well as educational campaigns to disseminate information regarding HPV infection and vaccine in order to increase the adherence and to reduce the infections.

Understanding barriers related with vaccine recommendation are necessary when considering the development of interventions that focus on increasing the vaccination. The most common reason cited by PCPs for not recommending HPV vaccine for boys was that in their Region for this group it was not free of charge, and this finding has important implications for HPV vaccine policy, because there was a policy barrier to recommend the vaccine. Indeed, at the time of the survey, HPV vaccine was provided free of charge to boys in nine Regions (Friuli-Venezia Giulia, Trentino-Alto Adige, Veneto, Liguria, Sardinia, Molise, Apulia, Calabria, Sicily). In addition, other reasons affecting HPV vaccine non-recommendation to boys were concerns about vaccine safety and efficacy. This is surprising because the safety and the efficacy, of the HPV vaccine have been determined in multiple studies [32–34] and there is accumulating evidence that adverse events are rare [35,36]. However, similar concerns about the usefulness of vaccines were noted in previous studies among pediatric health care professionals [30,37].

A main objective of the present investigation was to identify factors affecting the behavior of the PCPs regarding HPV vaccination. Indeed, understanding the factors associated with the recommendation of the HPV vaccine to boys and girls is critical because the recommendation of the PCPs is a key factor in their decision to get vaccinated. Findings from the current analysis indicate that the variable working in a Region where HPV immunization was actively recommended and provided free of charge to 11–12 year old boys is a robust predictor of vaccine recommendation by the PCPs to this group. Indeed, as expected, PCPs working in these geographic areas were more likely to recommend the HPV vaccine. The findings of the present study also indicate that personal beliefs about vaccine safety and efficacy were positively associated with vaccination recommendation either for boys or girls aged 11–12 years. Furthermore, not surprisingly and consistent with previous studies, scientific journals and educational courses/meetings were the principal information source that the PCPs trusted [38–41]. Along with earlier research, this survey suggest that the advice of scientific journals is crucial in performing appropriate practice among PCPs [42]. As a specific example of this, it has been found that the PCPs who obtain vaccine information from this source were more likely to always recommend the HPV vaccine to 11–12 year old girls than those who did not use such source, supporting the impact that scientific journals have in the decision making.

It is important to acknowledge that certain limitations need to be noted in the interpretation of the results of this study. First, since this was a cross-sectional survey, the analysis can only provide evidence of statistical association between explanatory variables and the different outcomes of interest and it is difficult to demonstrate temporal relationship. Second, the intrinsic problem associated with the use of questionnaire with self-reported information and thus the study may be subject to declaration or desirability biases. Social desirability may have influenced the sample and the participants may have responded to questions regarding their practice in a socially desirable way, even though questionnaire was anonymous and the items were carefully framed to avoid a judgmental tone. Third, it is possible that PCPs engaged in HPV vaccination or who had a positive attitude regard vaccination were more likely to respond to the survey than those who did not share these attitudes. These biases would have led to an overestimation of the number of PCPs who recommend HPV vaccination. Fourth, the response rate was modest, but superior to other studies with similar methodology [43–46] and it is well known that Web surveys have a lower response rate than those conducted with other methods [47,48]. Moreover, demographic characteristics of the respondents were similar to those of the non-respondents. This study also has important strengths. This is the first study to investigate PCPs attitude and practice towards HPV vaccination using a nationally representative Italian sample, with similar characteristics of the Italian PCPs [49], thereby proving important information regarding a population previously not addressed in the literature, it is possible to provide generalizable results while adjusting for potential confounders.

In conclusion, the current results highlight the importance that PCPs address barriers to HPV vaccination promotion and employ evidence-based educational strategies that can facilitate vaccine acceptability for adolescents in order to achieve a better coverage and to reduce the morbidities and mortality of diseases associated with HPV.

## Previous presentations

Preliminary findings from this study have been presented at the Annual Conference of the European Public Health Association, Stockholm, 1–4 November, 2017.

## Supporting information

S1 File Questionario.docx. Questionnaire in Italian.
(DOCX)

S2 File Questionnaire.docx. Questionnaire in English.
(DOCX)

S3 File Dataset.xlsx. Data file.
(XLSX)
