# Supplementary material for: Primary care pediatricians’ attitudes and practice towards HPV vaccination: A nationwide survey in Italy
Source: PLoS One. 2018 Mar 29;13(3):e0194920. doi: 10.1371/journal.pone.0194920 (PMC5875794; doi:10.1371/journal.pone.0194920)
Supplement: S1 File — Questionnaire in Italian. (DOCX) [file pone.0194920.s001.docx]

**QUESTIONARIO**

**A. INFORMAZIONI ANAGRAFICHE E PROFESSIONALI**

**Le domande in questa sezione mirano a raccogliere alcuni Suoi dati anagrafici e professionali.**

**A1.** Lei è Maschio o Femmina? □ Maschio □ Femmina

**A2.** Qual è la Sua età? ______

**A3.** In quale anno ha conseguito la laurea? _______

**A4.** In quale Regione svolge la Sua attività di PLS?________________________

**A5.** Da quanto tempo svolge la Sua attività di PLS (anni)?________

**A6.** Come svolge la Sua attività di PLS? □ da solo □ in gruppo (indicare con quanti altri PLS) _________

**A7.** Quanti pazienti ha a Suo carico?________________________

**A8.** Quante ore lavora in una settimana?__________________________________

**A9**. Quanti maschi tra 11 e 12 anni ha a Suo carico? ____________

**A10**. Quanti maschi tra 13 e 18 anni ha a Suo carico? ____________

**A11**. Quanti femmine tra 11 e 12 anni ha a Suo carico? ____________

**A12**. Quanti femmine tra 13 e 18 anni ha a Suo carico? ____________

**B. ATTITUDINI**

**Le domande in questa sezione mirano a valutare le Sue attitudini in tema di vaccinazione anti-HPV.**

**B1.** Secondo Lei, in una scala da 1 a 10, quanto è efficace la vaccinazione anti-HPV nelle femmine tra 11 e 18 anni?

| **non efficace** | **1** | **2** | **3** | **4** | **5** | **6** | **7** | **8** | **9** | **10** | **molto efficace** |
| --- | --- | --- | --- | --- | --- | --- | --- | --- | --- | --- | --- |

**B2.** Secondo Lei, in una scala da 1 a 10, quanto è efficace la vaccinazione anti-HPV nei maschi tra 11 e 18 anni?

| **non efficace** | **1** | **2** | **3** | **4** | **5** | **6** | **7** | **8** | **9** | **10** | **molto efficace** |
| --- | --- | --- | --- | --- | --- | --- | --- | --- | --- | --- | --- |

**B3.** Secondo Lei, in una scala da 1 a 10, quanto è sicuro il vaccino anti-HPV per i maschi e le femmine?

| **non sicuro** | **1** | **2** | **3** | **4** | **5** | **6** | **7** | **8** | **9** | **10** | **molto sicuro** |
| --- | --- | --- | --- | --- | --- | --- | --- | --- | --- | --- | --- |

**C. COMPORTAMENTI**

**Le domande in questa sezione mirano a valutare i Suoi comportamenti in tema di vaccinazione anti-HPV.**

**C1.** Parla dell’infezione da HPV, delle patologie correlate e della vaccinazione anti-HPV ai Suoi pazienti tra 11 e 18 anni o ai loro genitori?

Mai Raramente Talvolta Spesso Sempre

**C2.** Raccoglie informazioni sulle abitudini sessuali (numero di partner, frequenza di rapporti, etc.) dei Suoi pazienti tra 11 e 18 anni?

Mai Raramente Talvolta Spesso Sempre

**C3.** Raccoglie informazioni sull’utilizzo del preservativo fra i Suoi pazienti tra 11 e 18 anni?

Mai Raramente Talvolta Spesso Sempre

**C4.** Raccomanda la vaccinazione anti-HPV ai Suoi pazienti maschi tra 11 e 12 anni?

| **mai raramente talvolta**  **Per quale motivo?** (anche più di una risposta) |  | **spesso sempre**  **Per quale motivo?** (anche più di una risposta) |
| --- | --- | --- |
|  |  |  |
| □ Il vaccino è utile solo nel genere femminile |  | □ Il vaccino è sicuro |
| □ Preoccupazioni sulla sicurezza del vaccino |  | □ Il vaccino è efficace |
| □ Dubbi sull’efficacia del vaccino |  | □ Il vaccino può prevenire lesioni precancerose HPV-correlate |
| □ Carenza di informazioni sul vaccino |  | □ Il vaccino può prevenire i tumori HPV-correlati |
| □ Nella mia Regione non è attiva la campagna vaccinale anti-HPV nei maschi |  | □ Nella mia Regione è attiva la campagna vaccinale anti-HPV nei maschi |
| □ Mancanza di tempo per discutere dell’infezione da HPV con pazienti e genitori |  | □ Altro (specificare)______________________________ |
| □ La vaccinazione potrebbe incoraggiare a iniziare la attività sessuale |  |  |
| □ La vaccinazione potrebbe incoraggiare comportamenti sessuali a rischio |  |  |
| □ Genitori contrari alla vaccinazione |  |  |
| □ Altro (specificare)_________________________________________ |  |  |

**[se la risposta è “mai” andare a C6]**

**C5.** Tra i Suoi pazienti maschi tra 11 e 12 anni ai quali Lei raccomanda la vaccinazione anti-HPV, c’è qualcuno che non si vaccina?

| **No** | **Sì, per quale motivo?** (anche più di una risposta) |
| --- | --- |
|  | 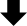 |
|  | □ Scarsa fiducia sulla efficacia del vaccino |
|  | □ Timore di effetti collaterali del vaccino |
|  | □ Non conoscenza dell’infezione da HPV |
|  | □ Convinzione che l’infezione da HPV non sia grave |
|  | □ Convinzione di non essere un soggetto a rischio |
|  | □ Il vaccino è costoso |
|  | □ Il vaccino è utile solo nel genere femminile |
|  | □ Contrari alle vaccinazioni |
|  | □ Altro (specificare)__________________________ |

**C6.** Raccomanda la vaccinazione anti-HPV ai Suoi pazienti maschi tra 13 e 18 anni non precedentemente vaccinati?

| **mai raramente talvolta**  **Per quale motivo?** (anche più di una risposta) |  | **spesso sempre**  **Per quale motivo?** (anche più di una risposta) |
| --- | --- | --- |
|  |  |  |
| □ Il vaccino è utile solo nel genere femminile |  | □ Il vaccino è sicuro |
| □ Preoccupazioni sulla sicurezza del vaccino |  | □ Il vaccino è efficace |
| □ Dubbi sull’efficacia del vaccino |  | □ Il vaccino può prevenire lesioni precancerose HPV-correlate |
| □ Mancanza di informazioni sul vaccino |  | □ Il vaccino può prevenire i tumori HPV-correlati |
| □ Nella mia Regione non è attiva la campagna vaccinale anti-HPV nei maschi |  | □ Nella mia Regione è attiva la campagna vaccinale anti-HPV nei maschi |
| □ Mancanza di tempo per discutere dell’infezione da HPV con i pazienti e i genitori |  | □ Altro (specificare)______________________________ |
| □ La vaccinazione potrebbe incoraggiare a iniziare la attività sessuale |  |  |
| □ La vaccinazione potrebbe incoraggiare comportamenti sessuali a rischio |  |  |
| □ Genitori contrari alla vaccinazione |  |  |
| □ Altro (specificare)_________________________________________ |  |  |

**[se la risposta è “mai” andare a C8]**

**C7.** Tra i Suoi pazienti maschi tra 13 e 18 anni ai quali Lei raccomanda la vaccinazione anti-HPV, c’è qualcuno che non si vaccina?

| **No** | **Sì, per quale motivo?** (anche più di una risposta) |
| --- | --- |
|  | 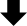 |
|  | □ Scarsa fiducia sulla efficacia del vaccino |
|  | □ Timore di effetti collaterali del vaccino |
|  | □ Non conoscenza dell’infezione da HPV |
|  | □ Convinzione che l’infezione da HPV non sia grave |
|  | □ Convinzione di non essere un soggetto a rischio |
|  | □ Il vaccino è costoso |
|  | □ Il vaccino è utile solo nel genere femminile |
|  | □ Contrari alle vaccinazioni |
|  | □ Altro (specificare)__________________________ |

**C8.** Raccomanda la vaccinazione anti-HPV alle Sue pazienti femmine tra 11 e 12 anni?

| **mai raramente talvolta**  **Per quale motivo?** (anche più di una risposta) |  | **spesso sempre**  **Per quale motivo?** (anche più di una risposta) |
| --- | --- | --- |
|  |  |  |
| □ Preoccupazioni sulla sicurezza del vaccino |  | □ Il vaccino è sicuro |
| □ Preoccupazioni sull’efficacia del vaccino |  | □ Il vaccino è efficace |
| □ Mancanza di informazioni sul vaccino |  | □ Il vaccino può prevenire le lesioni precancerose HPV-correlate |
| □ Nella mia Regione non è attiva una campagna vaccinale anti-HPV nelle femmine |  | □ Il vaccino può prevenire i tumori HPV-correlati |
| □ Mancanza di tempo per discutere dell’infezione HPV con i pazienti e i genitori |  | □ Nella mia Regione è attiva una campagna vaccinale anti-HPV nelle femmine |
| □ La vaccinazione potrebbe incoraggiare a iniziare la attività sessuale |  | □ Altro (specificare)______________________________ |
| □ La vaccinazione potrebbe incoraggiare comportamenti sessuali a rischio |  |  |
| □ Genitori contrari alla vaccinazione |  |  |
| □ Altro (specificare)_________________________________________ |  |  |

**[se la risposta è “mai” andare a C10]**

**C9.** Tra le Sue pazienti femmine tra 11 e 12 anni alle quali Lei raccomanda la vaccinazione anti-HPV, c’è qualcuna che non si vaccina?

| **No** | **Sì, per quale motivo?** (anche più di una risposta) |
| --- | --- |
|  | 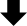 |
|  | □ Scarsa fiducia sulla efficacia del vaccino |
|  | □ Timore di effetti collaterali del vaccino |
|  | □ Non conoscenza dell’infezione da HPV |
|  | □ Convinzione che l’infezione da HPV non sia grave |
|  | □ Convinzione di non essere un soggetto a rischio |
|  | □ Il vaccino è costoso |
|  | □ Contrari alle vaccinazioni |
|  | □ Altro (specificare)__________________________ |

**C10.** Raccomanda la vaccinazione anti-HPV alle Sue pazienti femmine tra 13 e 18 anni non precedentemente vaccinate?

| **mai raramente talvolta**  **Per quale motivo?** (anche più di una risposta) |  | **spesso sempre**  **Per quale motivo?** (anche più di una risposta) |
| --- | --- | --- |
|  |  |  |
| □ Preoccupazioni sulla sicurezza del vaccino |  | □ Il vaccino è sicuro |
| □ Preoccupazioni sull’efficacia del vaccino |  | □ Il vaccino è efficace |
| □ Mancanza di informazioni sul vaccino |  | □ Il vaccino può prevenire le lesioni precancerose HPV-correlate |
| □ Nella mia Regione non è attiva una campagna vaccinale anti-HPV nelle femmine |  | □ Il vaccino può prevenire i tumori HPV-correlati |
| □ Mancanza di tempo per discutere dell’infezione HPV con i pazienti e i genitori |  | □ Nella mia Regione è attiva una campagna vaccinale anti-HPV nelle femmine |
| □ La vaccinazione potrebbe incoraggiare a iniziare la attività sessuale |  | □ Altro (specificare)______________________________ |
| □ La vaccinazione potrebbe incoraggiare comportamenti sessuali a rischio |  |  |
| □ Genitori contrari alla vaccinazione |  |  |
| □ Altro (specificare)_________________________________________ |  |  |

**[se la risposta è “mai” andare alla sezione D]**

**C11.** Tra le Sue pazienti femmine tra 13 e 18 anni alle quali Lei raccomanda la vaccinazione anti-HPV, c’è qualcuna che non si vaccina?

| **No** | **Sì, per quale motivo?** (anche più di una risposta) |
| --- | --- |
|  | 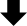 |
|  | □ Scarsa fiducia sulla efficacia del vaccino |
|  | □ Timore di effetti collaterali del vaccino |
|  | □ Non conoscenza dell’infezione da HPV |
|  | □ Convinzione che l’infezione da HPV non sia grave |
|  | □ Convinzione di non essere un soggetto a rischio |
|  | □ Il vaccino è costoso |
|  | □ Contrari alle vaccinazioni |
|  | □ Altro (specificare)__________________________ |

**D. INFORMAZIONI**

**Le domande in questa sezione mirano a valutare le fonti di informazione in tema di infezione da HPV e vaccinazione.**

**D1.** Da quali fonti riceve informazioni sulla infezione da HPV? (anche più di una risposta)

Nessuna Riviste scientifiche Mass-media Congressi/convegni Colleghi

 Altro (specificare) ______________

**D2.** Da quali fonti riceve informazioni sulla vaccinazione anti-HPV? (anche più di una risposta)

Nessuna Riviste scientifiche Mass-media Congressi/convegni Colleghi

 Altro (specificare) ______________

**D3.** Ritiene di aver bisogno di ulteriori informazioni sulla infezione da HPV? No Si

**D4.** Ritiene di aver bisogno di ulteriori informazioni sulla vaccinazione anti-HPV? No Si

**Il questionario è finito. Se vuole aggiungere qualcosa, utilizzi lo spazio sottostante**

**______________________________________________________________________________________________________________________________________________________________________________________________________**

**GRAZIE PER AVER PARTECIPATO ALL’INDAGINE**
