# Supplementary material for: Primary care pediatricians’ attitudes and practice towards HPV vaccination: A nationwide survey in Italy
Source: PLoS One. 2018 Mar 29;13(3):e0194920. doi: 10.1371/journal.pone.0194920 (PMC5875794; doi:10.1371/journal.pone.0194920)
Supplement: S2 File — Questionnaire in English. (DOCX) [file pone.0194920.s002.docx]

**QUESTIONNAIRE**

**A. DEMOGRAPHIC AND PRACTICE CHARACTERISTICS**

**This section is designed to gather information about your socio-demographic and practice characteristics**

**A1.** What is your gender? □ Male □ Female

**A2.** How old were you on your last birthday? ________

**A3.** In what year did you obtained your Medical Degree? _________

**A4.** In which Region is your practice setting?________________________

**A5.** How long have you been in practice (years)?________

**A6.** Which is your type of practice? □ solo □ group (indicate the number of your Colleagues) _________

**A7.** How many patients do you have?________________________

**A8.** How many hours do you spend in patient care in a typical week?__________________________________

**A9**. How many male patients of 11-12 years do you have? ____________

**A10**. How many male patients of 13-18 years do you have? ____________

**A11**. How many female patients of 11-12 years do you have? ______

**A12**. How many female patients of 13-18 years do you have? ______

**B. ATTITUDES**

**This section is designed to explore your attitudes towards HPV vaccination**

**B1.** How effective do you think the HPV vaccination is for 11-18 year old girls?

| **Not effective** | **1** | **2** | **3** | **4** | **5** | **6** | **7** | **8** | **9** | **10** | **Very effective** |
| --- | --- | --- | --- | --- | --- | --- | --- | --- | --- | --- | --- |

**B2.** How effective do you think the HPV vaccination is for 11-18 year old boys?

| **Not effective** | **1** | **2** | **3** | **4** | **5** | **6** | **7** | **8** | **9** | **10** | **Very effective** |
| --- | --- | --- | --- | --- | --- | --- | --- | --- | --- | --- | --- |

**B3.** How safe do you think is the HPV vaccination for boys and girls?

| **Not safe** | **1** | **2** | **3** | **4** | **5** | **6** | **7** | **8** | **9** | **10** | **Very safe** |
| --- | --- | --- | --- | --- | --- | --- | --- | --- | --- | --- | --- |

**C. PRACTICE**

**This section is designed to gather information about your practice towards HPV vaccination**

**C1.** How often do you talk about HPV infection and HPV related diseases with your patients of 11-18 years or their parents?

Never Rarely Sometimes Often Always

**C2.** How often do you collect information about sexual habits (number of partners and of sexual intercourses) of your patients of 11-18 years?

 Never Rarely Sometimes Often Always

**C3.** How often do you collect information about condom use of your patients of 11-18 years?

 Never Rarely Sometimes Often Always

**C4.** How often do you recommend HPV vaccination to male adolescents of 11-12 years?

| **never rarely sometimes**  **For which reason?** (more than one answer is allowed) |  | **often always**  **For which reason?** (more than one answer is allowed) |
| --- | --- | --- |
|  |  |  |
| □ Vaccine is useful only for female patients |  | □ Vaccine is safe |
| □ Concerns about vaccine safety |  | □ Vaccine is effective |
| □ Concerns about vaccine efficacy |  | □ Vaccine can prevent HPV-related precancerous lesions |
| □ Lack of information about the vaccine |  | □ Vaccine can prevent HPV-related cancers |
| □ I work in a Region where the vaccination was not actively recommended and provided free of charge to boys |  | □ I work in a Region where the vaccination was actively recommended and provided free of charge to boys |
| □ Lack of time to talk with patients and parents about HPV infection |  | □ Other (please specify)___________________________ |
| □ Vaccination could increase sexual activities |  |  |
| □ Vaccination could increase the high-risk sexual behaviors |  |  |
| □ Parents have an objection to the administration of vaccines |  |  |
| □ Other (please specify)_______________________________________ |  |  |

**(if You answered “never”, please go to question C6.)**

**C5**. Among your male patients of 11-12 years to whom you have recommended the HPV vaccine, there are patients who refuse it?

| **No** | **Yes, for which reason?** (more than one answer is allowed) |
| --- | --- |
|  | 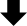 |
|  | □ Concerns regarding the vaccine efficacy |
|  | □ Concerns regarding the adverse side effects |
|  | □ Non enough knowledge about HPV infection |
|  | □ Belief that HPV infection is not severe |
|  | □ Belief of being not at risk for HPV infection |
|  | □ Vaccination costs |
|  | □ Vaccine is useful only for female patients |
|  | □ Objection to the administration of vaccines |
|  | □ Other (please specify)_______________________________ |

**C6.** How often do you recommend HPV vaccination to male adolescents of 13-18 years not previously vaccinated?

| **never rarely sometimes**  **For which reason?** (more than one answer is allowed) |  | **often always**  **For which reason?** (more than one answer is allowed) |
| --- | --- | --- |
|  |  |  |
| □ Vaccine is useful only for female patients |  | □ Vaccine is safe |
| □ Concerns about vaccine safety |  | □ Vaccine is effective |
| □ Concerns about vaccine efficacy |  | □ Vaccine can prevent HPV-related precancerous lesions |
| □ Lack of information about the vaccine |  | □ Vaccine can prevent HPV-related cancers |
| □ I work in a Region where the vaccination was not actively recommended and provided free of charge to boys |  | □ I work in a Region where the vaccination was actively recommended and provided free of charge to boys |
| □ Lack of time to talk with patients and parents about HPV infection |  | □ Other (please specify)___________________________ |
| □ Vaccination could increase sexual activities |  |  |
| □ Vaccination could increase the high-risk sexual behaviors |  |  |
| □ Parents have an objection to the administration of vaccines |  |  |
| □ Other (please specify)_______________________________________ |  |  |

**(if You answered “never”, please go to question C8.)**

**C7.** Among your male patients of 13-18 years to whom you have recommended the HPV vaccine, there are patients who refuse it?

| **No** | **Yes, for which reason?** (more than one answer is allowed |
| --- | --- |
|  | 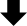 |
|  | □ Concerns regarding the vaccine efficacy |
|  | □ Concerns regarding the adverse side effects |
|  | □ Non enough knowledge about HPV infection |
|  | □ Belief that HPV infection is not severe |
|  | □ Belief of being not at risk for HPV infection |
|  | □ Vaccination costs |
|  | □ Vaccine is useful only for female patients |
|  | □ Objection to the administration of vaccines |
|  | □ Other (please specify)_______________________________ |

**C8.** How often do you recommend HPV vaccination to female adolescents of 11-12 years?

| ** never rarely sometimes**  **For which reason?** (more than one answer is allowed) |  | ** often always**  **For which reason?** (more than one answer is allowed) |
| --- | --- | --- |
|  |  |  |
| □ Concerns about vaccine safety |  | □ Vaccine is safe |
| □ Concerns about vaccine efficacy |  | □ Vaccine is effective |
| □ Lack of information about the vaccine |  | □ Vaccine can prevent HPV-related precancerous lesions |
| □ I work in a Region where the vaccination was not actively recommended and provided free of charge to girls |  | □ Vaccine can prevent HPV-related cancers |
| □ Lack of time to talk with patients and parents about HPV infection |  | □ I work in a Region where the vaccination was actively recommended and provided free of charge to girls |
| □ Vaccination could increase sexual activities |  | □ Other (please specify)___________________________ |
| □ Vaccination could increase the high-risk sexual behaviors |  |  |
| □ Parents have an objection to the administration of vaccines |  |  |
| □ Other (please specify)_______________________________________ |  |  |

**(if You answered “never”, please go to question C10.)**

**C9.** Among your female patients of 11-12 years to whom you have recommended the HPV vaccine, there are patients who refuse it?

| **No** | **Yes, for which reason?** (more than one answer is allowed) |
| --- | --- |
|  | 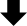 |
|  | □ Concerns regarding the vaccine efficacy |
|  | □ Concerns regarding the adverse side effects |
|  | □ Non enough knowledge about HPV infection |
|  | □ Belief that HPV infection is not severe |
|  | □ Belief of being not at risk for HPV infection |
|  | □ Vaccination costs |
|  | □ Objection to the administration of vaccines |
|  | □ Other (please specify)_______________________________ |

**C10.** How often do you recommend HPV vaccination to female adolescents of 13-18 years not previously vaccinated?

| ** never rarely sometimes**  **For which reason?** (more than one answer is allowed) |  | ** often always**  **For which reason?** (more than one answer is allowed) |
| --- | --- | --- |
|  |  |  |
| □ Concerns about vaccine safety |  | □ Vaccine is safe |
| □ Concerns about vaccine efficacy |  | □ Vaccine is effective |
| □ Lack of information about the vaccine |  | □ Vaccine can prevent HPV-related precancerous lesions |
| □ I work in a Region where the vaccination was not actively recommended and provided free of charge to girls |  | □ Vaccine can prevent HPV-related cancers |
| □ Lack of time to talk with patients and parents about HPV infection |  | □ I work in a Region where the vaccination was actively recommended and provided free of charge to girls |
| □ Vaccination could increase sexual activities |  | □ Other (please specify)___________________________ |
| □ Vaccination could increase the high-risk sexual behaviors |  |  |
| □ Parents have an objection to the administration of vaccines |  |  |
| □ Other (please specify)_______________________________________ |  |  |

**(if You answered “never”, please go to section D.)**

**C11.** Among your female patients of 13-18 years to whom you have recommended the HPV vaccine, there are patients who refuse it?

| **No** | **Yes, for which reason?** (more than one answer is allowed) |
| --- | --- |
|  | 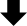 |
|  | □ Concerns regarding the vaccine efficacy |
|  | □ Concerns regarding the adverse side effects |
|  | □ Non enough knowledge about HPV infection |
|  | □ Belief that HPV infection is not severe |
|  | □ Belief of being not at risk for HPV infection |
|  | □ Vaccination costs |
|  | □ Objection to the administration of vaccines |
|  | □ Other (please specify)_______________________________ |

**D. INFORMATION**

**The section is designed to explore your sources of information towards HPV infection and vaccination**

**D1.** From which of the following sources do you receive information about HPV infection? (more than one answer is allowed)

None Scientific Journals Mass-media  Educational courses/Meetings Colleagues

 Other (please specify) ____________

**D2.** From which of the following sources do you receive information about HPV vaccination? (more than one answer is allowed)

None Scientific Journals Mass-media  Educational courses/Meetings Colleagues

 Other (please specify) ____________

**D3.** Do you feel you need more information about HPV infection? No Yes

**D4.** Do you feel you need more information about HPV vaccination? No Yes

**The questionnaire is finished. If you want to add something, use the space below**

**______________________________________________________________________________________________________________________________________________________________________________________________________**

**THANK YOU FOR PARTICIPATING IN THE SURVEY**
